# A 6-Month Home-Based Functional Electrical Stimulation Program for Foot Drop in a Post-Stroke Patient: Considerations on a Time Course Analysis of Walking Performance

**DOI:** 10.3390/ijerph19159204

**Published:** 2022-07-27

**Authors:** Romain David, Maxime Billot, Etienne Ojardias, Bernard Parratte, Manuel Roulaud, Amine Ounajim, Frédéric Louis, Hachemi Meklat, Philippe Foucault, Christophe Lombard, Anne Jossart, Laura Mainini, Martin Lavallière, Lisa Goudman, Maarten Moens, Davy Laroche, Marjorie Salga, François Genêt, Jean-Christophe Daviet, Anaick Perrochon, Maxence Compagnat, Philippe Rigoard

**Affiliations:** 1 PRISMATICS Lab (Predictive Research in Spine/Neuromodulation Management and Thoracic Innovation/Cardiac Surgery), Poitiers University Hospital, 86000 Poitiers, France; romain-david@hotmail.fr (R.D.); bernard.parratte@univ-fcomte.fr (B.P.); manuel.roulaud@chu-poitiers.fr (M.R.); amine.ounajim@chu-poitiers.fr (A.O.); philippe.rigoard@chu-poitiers.fr (P.R.); 2 Department of Physical and Rehabilitation Medicine, Poitiers University Hospital, 86000 Poitiers, France; anne.jossart@chu-poitiers.fr (A.J.); laura.mainini@chu-poitiers.fr (L.M.); 3 Physical Medicine and Rehabilitation Department, University Hospital of Saint-Etienne, 42270 Saint-Etienne, France; ojardiasetienne@gmail.com; 4 Department of Physical and Rehabilitation Medicine le Grand Feu, Rue de la Verrerie, 79000 Niort, France; flouis@melioris.fr; 5 Department of Physical and Rehabilitation Medicine Richelieu, Rue Philippe-Vincent, 17028 La Rochelle, France; hachemi.meklat@croix-rouge.fr (H.M.); philippe.foucault@croix-rouge.fr (P.F.); christophe.lombard@croix-rouge.fr (C.L.); 6 Module de Kinésiologie, Département des Sciences de la Santé, CISD, & Lab BioNR, Université du Québec à Chicoutimi, Chicoutimi, QC G7H 2B1, Canada; martin_lavalliere@uqac.ca; 7 Department of Neurosurgery, Universitair Ziekenhuis Brussel, Laarbeeklaan 101, 1090 Brussels, Belgium; lisa.goudman@gmail.com (L.G.); mtmoens@gmail.com (M.M.); 8 STIMULUS Consortium (reSearch and TeachIng neuroModULation Uz bruSsel), Vrije Universiteit Brussel, Laarbeeklaan 103, 1090 Brussels, Belgium; 9 Research Foundation—Flanders (FWO), 1090 Brussels, Belgium; 10 Department of Radiology, Universitair Ziekenhuis Brussel, Laarbeeklaan 101, 1090 Brussels, Belgium; 11 INSERM UMR1093 Cognition, Action and Sensorimotor Plasticity Research Unit, UFR des Sciences du Sport, Université Bourgogne Franche-Comté, 21078 Dijon, France; davy.laroche@chu-dijon.fr; 12 INSERM, Centre d’Investigation Clinique 1432, Module Plurithematique, Plateforme d’Investigation Technologique, CHU Dijon-Bourgogne, Centre d’Investigation Clinique, Module Plurithématique, Plateforme d’Investigation Technologique, 21079 Dijon, France; 13 UPOH (Unité Péri Opératoire du Handicap, Perioperative Disability Unit), Physical and Rehabilitation Medicine Department, Raymond-Poincaré Hospital, Assistance Publique—Hôpitaux de Paris (AP-HP), 92380 Garches, France; marjorie.salga@aphp.fr (M.S.); francois.genet@aphp.fr (F.G.); 14 Inserm U1179, END-ICAP (Handicap neuromusculaire: Physiopathologie, Biothérapie et Pharmacologie Appliquées), UFR Simone Veil—Santé, Versailles Saint-Quentin-en-Yvelines University (UVSQ), 78180 Montigny-le-Bretonneux, France; 15 HAVAE UR20217 (Handicap, Ageing, Autonomy, Environment), University of Limoges, 87000 Limoges, France; jean-christophe.daviet@unilim.fr (J.-C.D.); anaick.perrochon@unilim.fr (A.P.); maxence.compagnat@unilim.fr (M.C.); 16 Department of Physical Medicine and Rehabilitation, University Hospital Center of Limoges, 87000 Limoges, France; 17 Department of Neuro-Spine & Neuromodulation, Poitiers University Hospital, 86000 Poitiers, France; 18 Prime Institute UPR 3346, CNRS, ISAE-ENSMA (Institut Supérieur de l’Aéronautique et de l’Espace—École Nationale Supérieure de Mécanique et d’Aérotechnique Poitiers Futuroscope), University of Poitiers, 86000 Poitiers, France

**Keywords:** foot drop, walking, rehabilitation, ankle-foot orthosis, mobility, functional electrical stimulation

## Abstract

Foot drop is a common disability in post-stroke patients and represents a challenge for the clinician. To date, ankle foot orthosis (AFO) combined with conventional rehabilitation is the gold standard of rehabilitation management. AFO has a palliative mechanical action without actively restoring the associated neural function. Functional electrical stimulation (FES), consisting of stimulation of the peroneal nerve pathway, represents an alternative approach. By providing an FES device (Bioness L-300, BIONESS, Valencia, CA, USA) for 6 months to a post-stroke 22-year-old woman with a foot drop, our goal was to quantify its potential benefit on walking capacity. The gait parameters and the temporal evolution of the speed were collected with a specific connected sole device (Feet Me^®^) during the 10-m walking, the time up and go, and the 6-minute walking tests with AFO, FES, or without any device (NO). As a result, the walking speed changes on 10-m were clinically significant with an increase from the baseline to 6 months in AFO (+0.14 m.s^−1^), FES (+0.36 m.s^−1^) and NO (+0.32 m.s^−1^) conditions. In addition, the speed decreased at about 4-min in the 6-minute walking test in NO and AFO conditions, while the speed increased in the FES conditions at baseline and after 1, 3, and 6 months. In addition to the walking performance improvement, monitoring the gait speed in an endurance test after an ecological rehabilitation training program helps to examine the walking performance in post-stroke patients and to propose a specific rehabilitation program.

## 1. Introduction

Gait impairments that are observed in 20–25% of stroke survivors [1,2], typically characterized by foot drop that is caused by a deficit of central common drive of dorsi-flexor muscles, represent a major challenge for rehabilitation [2,3]. The gold-standard care combines physical rehabilitation with passive ankle foot orthosis (AFO), a device with a substantial restriction of ankle movements which causes joint stiffness and possible sensory feedback loss without restoring any neural function [4]. While the AFO device allows limited ankle movement, functional electrical stimulation (FES), introduced 60 years ago [5], represents a worthwhile alternative that is likely to prevent these issues. FES consists of stimulation of the peroneal nerve pathway combined with (or without) voluntary contraction during the swing phase of walking [6]. 

To date, the literature has failed to provide evidence of FES superiority in comparison with AFO on walking performance [7]. A recent meta-analysis, including seven randomized controlled studies, showed that AFO produced the same therapeutic benefit on walking speed as FES [7]. Even though the authors reported moderate to low risk of bias, several concerns should be considered before burying FES permanently. Among the seven trials, one did not include post-stroke patients and cannot be further considered to determine FES efficacy in the post-stroke population. In the six remaining trials, the use of botulinium toxin before the trial was not mentioned, or the expected changes were not reported. In addition, three studies delivered FES in a supervised program with limited exposure to the therapy (20–40 min, 5 × week). The three remaining studies used FES daily at home for a short-to-long time period (6 to 30 weeks). While wearing the FES device could be considered as optimal to deliver the therapy, FES was applied during the chronic phase (>6 months post-stroke) in 55–60-year-old patients who had already used AFO in two out of the three studies. There is currently no consensus in the literature regarding the influence of age on gait recovery. More specifically while some studies have reported better recovery in younger patients [8,9,10], others have shown contrasting results [10,11]. In addition, age could largely influence the initial disability after stroke [8,10,12]. To date, the influence of age on walking recovery after a rehabilitation program with FES has yet to be examined and could be a positive predictive factor for successful outcomes.

By aiming to determine the potential beneficial effect of FES at home over a 6-month period, we delivered an FES device to a 22-year-old woman who suffered from a deficit of dorsi-flexor muscles following a stroke that had occurred 5 years earlier. Considering the long home care period and young age of the patient presenting limited dorsiflexion, we expected an improvement of the gait parameters that were assessed with connected sole before and after periods of 1, 3, and 6 months. This case will help clinicians and researchers to explore new directions of care pathways and evaluation, and to raise new perspectives.

## 2. Case Presentation

On May 2015, an 18-year-old woman, a high school student, and former basketball player, was admitted to Poitiers University Hospital and treated for a hemorrhagic stroke due to a left frontotemporal arteriovenous malformation. Severe left spastic hemiparesis and aphasia were found on examination as sequels. The Modified Ashworth Scale (MAS) revealed scores of 2 for the quadriceps, 1+ for the triceps surae muscle, and 2 for the tibialis anterior inducing varus foot. The patient reported no ankle pathology prior to her stroke. She was initially referred to a French rehabilitation center from 3 January to 15 March 2016, which led to limited walking recovery requiring the use of an assistive cane and inducing fatigue sensation [13] after a few meters. While the patient denied any AFO due to discomfort, she used an elastic lifter. She had a 450 m walking perimeter and was able to climb up and down stairs; she was not able to run. Thereafter, uncomfortable spasticity persisted and was treated with botulinum toxin injections. The last dose was injected in February 2018, and she underwent physical therapy twice a week. Physical therapy focusing on the lower limb consisted in exercises of equilibrium, stretching and walking, and in passive and active ankle dorsiflexion, knee extension, and hip adductions. On 8 April 2019, we offered to the 22-year-old woman the opportunity to test functional electric stimulation using an electronic orthosis (Bioness L-300, BIONESS, USA). After making the adjustments and teaching the patient how to use the device, she was able to wear it daily. The device was placed under the knee, about 2 cm from the head of the fibula in order to be as close as possible to the common fibular nerve. The objective was to set up functional rehabilitation with the FES at home, in an ecological situation over 6 months. She continued to receive physical therapy once a week during the 6-month period.

## 3. Investigation

Gait parameters were collected with a specific connected sole device, Feet Me^®^ (FeetMe^®^, Paris, France), that was placed inside the patient’s shoes allowing continuous analysis of the parameters of the walk. The gait parameter data were collected and stored within the insoles with an acquisition frequency of 140 Hz over the total duration of the tests. The speed was collected to characterize gait in three functional walking tests: the 10-m walking test (10-MWT), the time up and go (TUG) test, and the 6-minute walking test (6-MWT). In addition, the speed changes occurring during the 6-MWT were recorded. All of the tests were performed in three conditions: without any orthosis (NO), with AFO, and with FES. All the measurements were assessed before (M0) and after 1 (M1), 3 (M3), and 6 (M6) months. She had to walk 20 m with or without any device before each recording.

The wearing time of the FES device was reported on a daily diary. All the data were transferred to a secured web platform (FeetMe^®^ Mobility Dashboard, via FeetMe^®^) and then computed offline [14]. The calculation of the derivative and the sum of sensor signals were filtered by the Savitzky–Golay filter (please see [15] for details). The speed (in m.s^−1^) changes over time occurring during the 6-MWT were analyzed using locally estimated scatterplot smoothing (LOESS) with local polynomial regression. The minimal clinically important difference (MCID) was considered to evaluate the gait parameter evolution. 

## 4. Results

The wearing time of the FES device per day increased from 15 to 60 min during the first week, 1 to 4 h during the second week, and 4 to 10 h during the third week in accordance with the protocol for the use of the device. Over the following weeks, the patient wore the device all day long. The patient reported an increment of walking perimeter with a reduction of fatigue sensation, and a clinical assessment showed a decrease of varus foot without any adverse event such as burning, pain, or other uncomfortable sensations. Furthermore, she was able to run under supervision at M1 and without supervision from M3.

The 10-MWT walking speed changes were clinically significant (>0.14 m.s^−1^) [16] with a decrease from M0 to M3 in AFO and FES conditions (−0.32 m.s^−1^ and −0.29 m.s^−1^, respectively), and an increase from M0 to M6 in AFO and FES conditions (+0.14 m.s^−1^ and +0.36 m.s^−1^), without any clinically significant results in NO condition (Table 1).

Improvement of the TUG test was clinically significant (>28%) [17] at M6 compared to M0 in the AFO conditions (7.8 s vs. 2.8 s, +35%), while the increases in other conditions were not clinically significant.

**Figure 1:**
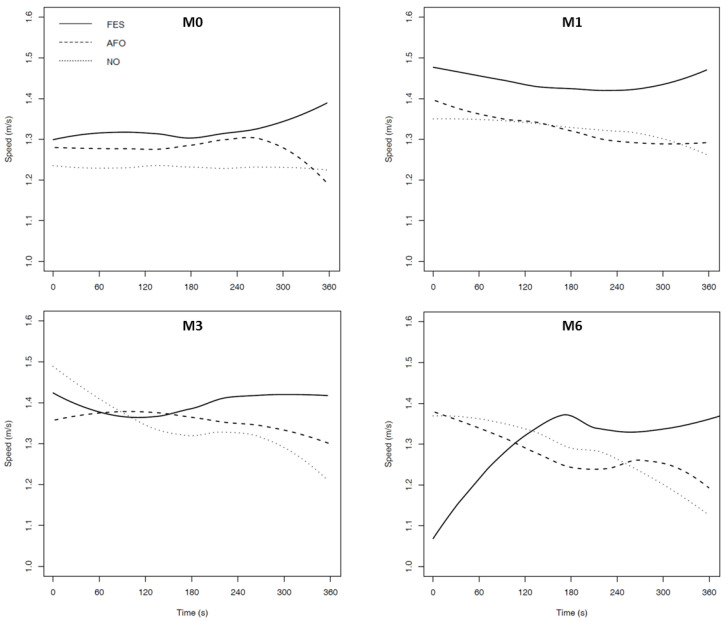
Gait speed evolution during the 6-MWT with NO (without any device), AFO (ankle-foot orthesis), and FES (functional electric stimulation) conditions at baseline (M0) and after 1 (M1), 3 (M3), and 6 (M6) months.

The 6-MWT distance increased from M0 to M1, M3, and M6 in NO, AFO, and FES conditions (Table 1), but the difference was not clinically significant (<28 m) [18,19]. On the other hand, the trajectories of the gait speed during the 6-MWT clearly showed that the speed decreased throughout the test in NO and AFO conditions, while the speed increased in the FES conditions at M0, M1, M3, and M6 (Figure 1). 

## 5. Discussion

This case provided evidence that the daily wearing of the FES device over a 6-month period can benefit post-stroke functional walking capacity. There are three main results that should be discussed. 

First, the walking speed decreased after 3 months and increased after 6 months. While there is no strong evidence in the literature, the long-term daily wearing of the FES device could be a basic requirement for a meaningful clinical outcome. Then again, is the ultimate clinical goal to improve walking performance in post-stroke rehabilitation? To date, functional rehabilitation programs have focused on walking performance, by assessing the walking capacity with a short-distance test (10 m) and/or endurance with long distance/time tests (400 m, 4–6 min walking test) [20,21]. Although walking speed is a crucial indicator to characterize functional mobility [22,23] and social participation [24,25], it is also limited by a restrictive view of the functional capacity of an individual. For instance, in clinical practice we have all encountered patients for whom walking speed did not meet the minimal clinically important difference after a functional rehabilitation program, while the walking duration was improved (walking capacity), delaying the neuromuscular fatigue effect [26] and the sensation of fatigue [13] for a day and dramatically enhancing the person’s quality of life. For the sake of the patient, it would seem advisable to replace walking performance by a general health status evaluation including the nature and localization of the stroke, walking capacity related to the goal of the patient, quality of life, social factors, psychological distress, pain, and the functional capacity that is drawn from the recent multidimensional and composite outcomes that are proposed to characterize general health status in chronic pain patients [27,28,29,30]. This approach could highlight the potential benefit that is achieved with the FES device masked behind the walking performance that is achieved after a rehabilitation program.

Second, the walking speed improvement in FES and AFO conditions seems not to be transferable to walking without any device. Although the patient was young, FES wearing occurred five years after the stroke episode. The chronic phase that characterized our case should be considered as an obstacle to FES benefit, notably regarding the ability to effectively walk without any device and in more ecological situations. Based on this finding and on the available literature, should we propose FES for every patient presenting with a deficit of common drive of the dorsi-flexors? The international stroke rehabilitation guidelines have established that early initiation of the rehabilitation program beyond the first week with the highest possible level of intensity provides variably greater recovery, depending on the nature and localization of the stroke [2], with greater cerebral plasticity observed in acute than in chronic stroke patients [31,32]. In a randomized controlled trial, Morone et al. [33] showed a greater improvement of walking speed (10 m) following a 5-week rehabilitation program with FES (20 sessions of 40 min) than AFO in 20 subacute post-stroke patients. In addition to the sub-acute and acute phase, the authors suggested the potential influence of age in predicting a positive clinical response to the therapy for a given patient. On the other hand, based on a selection of spatiotemporal and kinematic parameters, Chantraine et al. [34] proposed a classification of stroke patients indicating that FES should be targeted to patients with limited ankle dorsiflexion during the stance phase of walking. Taken together, these factors could be combined to determine a predictive approach that is related to positive response to FES in a post-stroke patient.

Third, the monitoring of gait speed during the 6-MWT enabled us to observe that the speed evolution profile is different with FES compared to the NO and AFO conditions [35]. Our results clearly showed a speed decrease with AFO or without any device at approximately 4 min, while the speed tends to increase in FES conditions. We prudently suggest that, in our case, FES delayed the onset of neuromuscular fatigue [26] and compensated for the fatigue that is shown to have occurred in the other conditions. This finding could help to delineate a personalized approach by developing specific programs related to the neuromuscular fatigue threshold that is observed in ecological situations, the objective being to delay this threshold. Accordingly, FES should be considered in the young acute/subacute/chronic stroke population and the old active acute/subacute/chronic stroke population presenting as a means of improving walking parameters in endurance effort, which could help to achieve the recommended physical activity [36,37], and substantially improve the quality of life and social participation [24,25]. This approach could also be reinforced by individualized coaching at home [38].

Replacing FES in the clinical context, FES prescription in a functional rehabilitation program is currently based on the available randomized controlled trial that was synthetized in a recent meta-analysis [7], showing that FES was inconsistently delivered in an inpatient for a duration of 3 to 12 weeks with sessions lasting between 15 and 40 min [4,33,39,40], or used in daily life at home for 8 to 26 weeks [41,42]. While FES in a hospital rehabilitation program limits the subject’s exposure to the therapy, its use at home might represent a better way to potentiate its effects. In a multicenter randomized controlled trial, Everaert et al. [41] reported significant improvement of walking performance (4-min and 10-m tests) after 6 weeks of daily use of the FES device in 69 chronic phase post-stroke patients, without any difference between the FES and AFO devices. With the implantable peroneal nerve stimulator device in 29 chronic phase post-stroke patients, Kottink et al. [42] did not observe any significant improvement of walking speed (10 m test) after 4, 8, 12, and 26 weeks of follow-up. Even though the literature has failed so far to show the added value of FES at home compared to a short inpatient rehabilitation program, home care should be further explored to potentiate the benefits of FES on walking performance and global health status, taking into account the patients’ characteristics [34].

While positive effects were reported after long-term use FES in daily living in one case report, some concerns remain. First, depending on the device, the stimulation settings more or less require intervention from an expert so as to deliver the adequate stimulation during the swing phase of walking. In addition, depending on the country, the device and related consumables (electrodes) may not be covered by the health insurance system, causing a significant extra cost for the patient in comparison with AFO and consequently limiting access to and diffusion of the therapy. Additional data are needed (i) to determine the effectiveness of FES on the patient’s general health as assessed with a composite multidimensional index that was developed with the machine learning approach; and (ii) to determine the potential supra-spinal, spinal, or muscular plasticity in an ecological long-term training program, and to delineate the cost-utility of the FES rehabilitation program compared to AFO via a medico-economic study.

## 6. Conclusions

Our case report showed that the walking performance benefits with FES that is worn daily over a long-term period of 6 months in a young adult presenting with a chronic post-stroke. Moreover, monitoring the gait speed in an endurance test should help to examine the walking performance rehabilitation in post-stroke patients and to delineate specific tailored rehabilitation programs. This approach needs to be investigated in randomized controlled trials to examine the cost-utility of the FES device in acute and subacute stroke patients. 

## Figures and Tables

**Table 1:** Gait parameters for the 10-m walking test (10-MWT), the 6-min walking test (6-MWT), and the time up and go (TUG) test in NO (without any device), AFO (ankle-foot orthesis) and FES (functional electric stimulation) conditions before (M0) and after 1 (M1), 3 (M3), and 6 (M6) months.

	M0	M1	M3	M6
10-MWT (speed in m.s^−1^)				
NO	1.28	1.31	1.29	1.60
AFO	1.44	1.34	1.12	1.58
FES	1.37	1.29	1.08	1.73
TUG (duration in seconds)				
NO	6.0	7.2	5.0	4.9
AFO	7.8	6.0	7.0	5.0
FES	6.0	6.0	5.0	4.6
6-MWT (distance in meters)				
NO	427.3	446.5	449.7	451.0
AFO	429.5	439.5	445.1	449.8
FES	459.0	472.3	454.5	462.8

## Data Availability

Data sharing is not applicable.
